# Numerical and experimental evaluation of ultrasound-assisted convection enhanced delivery to transfer drugs into brain tumors

**DOI:** 10.1038/s41598-022-23429-w

**Published:** 2022-11-11

**Authors:** Ahmad Boroumand, Mehrnoush Mehrarya, Ashkan Ghanbarzadeh-Dagheyan, Mohammad Taghi Ahmadian

**Affiliations:** 1 School of Mechanical Engineering, Sharif University of Technology, Azadi Ave., Tehran, Iran; 2 Protein Research Center, Shahid Beheshti University, Tehran, Iran

**Keywords:** Cancer therapy, CNS cancer, Biomedical engineering, Acoustics

## Abstract

Central Nervous System (CNS) malignant tumors are a leading cause of death worldwide with a high mortality rate. While numerous strategies have been proposed to treat CNS tumors, the treatment efficacy is still low mainly due to the existence of the Blood–Brain Barrier (BBB). BBB is a natural cellular layer between the circulatory system and brain extracellular fluid, limiting the transfer of drug particles and confining the routine treatment strategies in which drugs are released in the blood. Consequently, direct drug delivery methods have been devised to bypass the BBB. However, the efficiency of these methods is not enough to treat deep and large brain tumors. In the study at hand, the effect of focused ultrasound (FUS) waves on enhancing drug delivery to brain tumors, through ultrasound-assisted convection-enhanced delivery (UCED), has been investigated. First, brain mimicking gels were synthesized to mimic the CNS microenvironment, and the drug solution was injected into them. Second, FUS waves with the resonance frequency of 1.1 MHz were applied to the drug injected zone. Next, a finite element (FE) model was developed to evaluate the pre-existing equation in the literature for describing the drug delivery via acoustic streaming in brain tissue. Experimental results showed that the FUS transducer was able to enhance the drug volume distribution up to 500% relative to convection-enhanced delivery alone (CED). Numerical analysis showed that the FE model could replicate the experimental penetration depths with a mean difference value of less than 21%, and acoustic streaming plays a significant role in UCED. Therefore, the results of this study could open a new way to develop FE models of the brain to better evaluate the UCED and reduce the costs of conducting clinical and animal studies.

## Introduction

**Figure 1:**
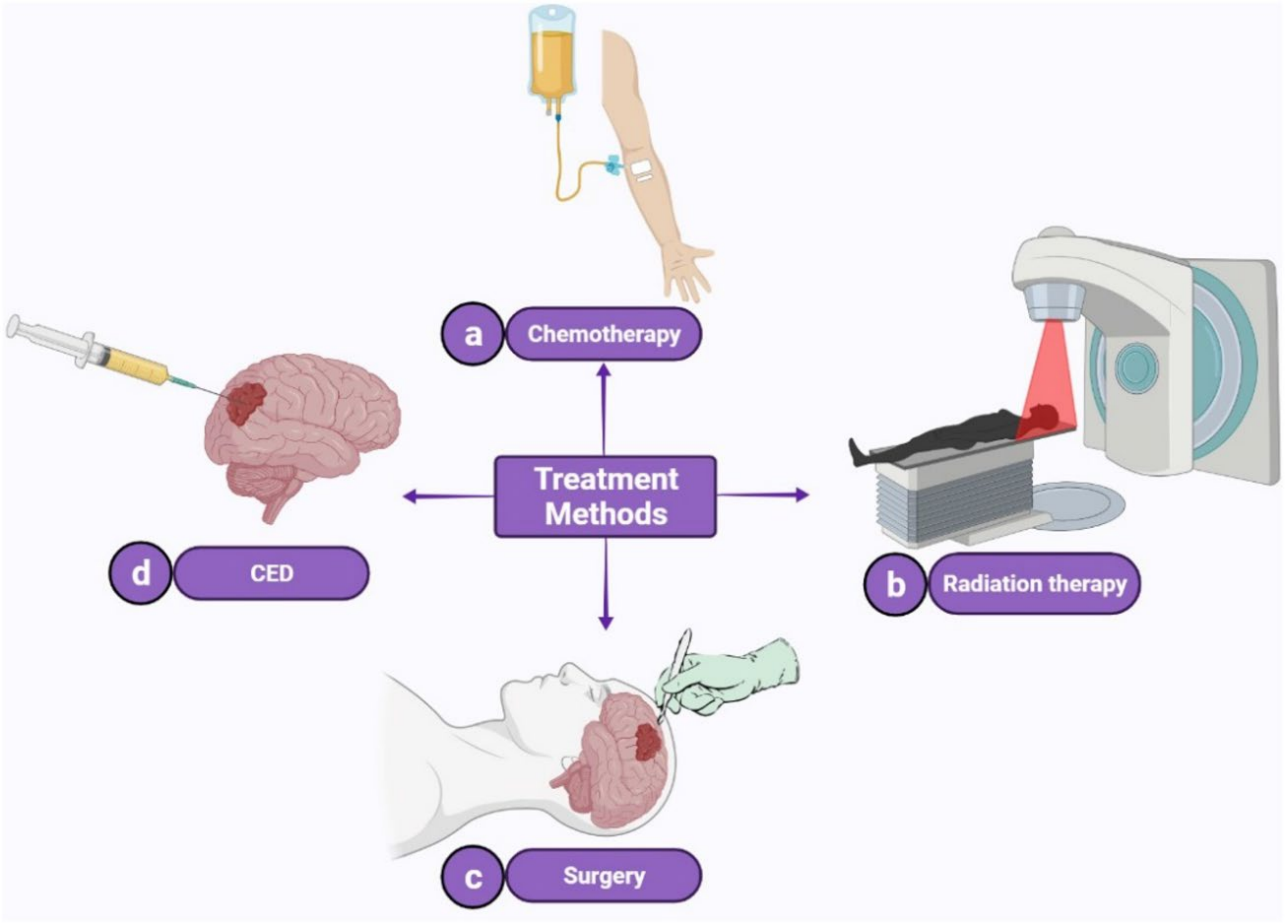
Different treatment strategies of brain tumors. In chemotherapy, the drug solution is injected into the blood circulatory system (**a**). In radiation therapy, X-ray or gamma-ray is radiated to the tumor cells to destroy them (**b**). Using surgical approaches, an expert opens the skull to remove the tumor (**c**). As for conventional drug delivery methods, the drug solution is injected directly to the tumor site via a thin catheter in CED (**d**).

A Central-Nervous-System (CNS) tumor begins when healthy cells within the brain or the spinal cord change and grow out of control, forming a mass. As reported by^1^, approximately 308,102 new cases of brain and other CNS tumors were diagnosed in the year 2020 worldwide, with an estimated 251,329 deaths. Various treatment methods have been implemented to cure CNS cancers. Among all treatment strategies, there are three major clinical approaches: chemotherapy, radiation therapy, surgery, (Fig. 1). Chemotherapy drugs can be administered orally or intravenously. Of the two, the latter delivers drugs immediately via the bloodstream to the tumor site (Fig. 1a). However, Blood–Brain Barrier (BBB) is a major obstacle to delivering therapeutics for the CNS disease, as it restricts many chemical compounds to penetrate the brain^2^. BBB possesses several layers composed of endothelial cells that separate systemic circulation from brain extracellular fluid^3^. These layers prevent chemical drugs entry into the target site and cause low treatment efficiency^4,5^.

Another treatment strategy is radiation therapy that uses the X-ray or gamma-ray to destroy tumor cells (Fig. 1b)^6^. This process takes weeks to shrink the tumor and it is usually implemented in combination with chemotherapy. The main problem associated with this method is the danger of damaging the surrounding healthy tissue that may cause severe problems for patients. Moreover, these rays themselves can damage DNA and cause cancer themselves^7–9^. Another invasive method is surgery, which is a process where the patient’s skull is opened to remove the entire or part of the tumor (Fig. 1c). Surgery is not an appropriate treatment method for all types of tumors. Furthermore, it may lead to brain damage^10^. Different novel methods have emerged to compensate for the shortcomings of the mentioned routine treatment strategies, and to use engineering methods for enhancing the results of treatment strategies^11,12^.

More novel methods are concentrated on bypassing the BBB. Convection-enhanced delivery (CED) has been introduced for the treatment of CNS cancer. In this method, a thin cannula is injected directly into the brain tissue to cross the BBB. The cannula’s tip is located at the tumor site, and then a pressure gradient with a syringe pump is applied to transfer drugs to the tumor (Fig. 1d). Creating a bulk flow inside the extracellular space, CED can deliver drugs at a depth in order of multi-centimeters into the brain tissue. However, this method can cause edema due to the concentration of drugs at the tip. Also, this method needs longer infusions or using more than one cannula in some cases^13^.

In recent years, using ultrasound waves in the field of biomedical engineering applications has drawn the scientists’ attention. Such waves are compatible with the human body and various effects can be extracted from them depending on the application and the area in the human body. Referring to those different effects, diverse techniques have used ultrasound waves to improve drug delivery to the brain or treat brain tumors, including thermal ablation, sonoporation, BBB disruption, and ultrasound-assisted drug delivery^11^. Among these techniques, ultrasound-assisted convection-enhanced delivery (UCED) has shown promising results in preclinical studies to improve drug delivery for treating brain tumors^14–19^. UCED is a method to overcome the shortcomings of CED and using the advantage of bypassing BBB. This treatment method is developed to improve the drug penetration depth, avoid edema, and better the drug distribution over the tumor tissue. In this method, first, the cannula’s tip is located at the tumor area, and a syringe pump injects the drug solution (refer to Subsect. “Drug injection system” for details) gently into that area. After the drug is loaded, an ultrasound transducer generates waves to help the drug solution penetrate deep into the tumor’s tissue.

Lewis, G. et al. used a focused ultrasound (FUS) transducer with a resonance frequency of 1.58 MHz to increase the penetration of Evans blue dye (EBD) into the equine brain, avian muscle, and agarose brain-mimicking gels. They reported that FUS waves could enhance drug penetration by 590% relative to diffusion. Moreover, applying FUS in combination with CED could enhance drug penetration by 880% relative to diffusion^15^. Liu, Y. et al. investigated the effect of a disk ultrasound transducer on drug penetration to porcine brain tissue in vitro and cynomolgus monkey in vivo. Different resonance frequencies were studied (85 kHz, 175 kHz and 1 MHz). They reported that an 85-kHz transducer could enhance the tissue's permeability 24 fold at an energy density of 1200 J/cm^2,17^.

In another study, Lewis, G.K. et al. investigated the effect of UCED on delivering drug solutions into rodent brain in an in-vivo setting. They fabricated a portable ultrasound system called transducer cannula assembly (TCA). The TCA was made up of an ultrasound transducer with a resonance frequency of 1.34 MHz and three 2400-mAh batteries. Four different experiments were conducted to investigate the effect of CED, CED with microbubbles, UCED and UCED with microbubbles. The results indicated that UCED could enhance the drug volume distribution up to 3.25 times relative to CED, and this number was 1.7 when UCED was used with microbubbles^16^. Mano, Y., et al. designed and fabricated a new device called ultrasound-facilitated delivery (UFD) system. A vast range of frequencies and voltages were studied to investigate the ability of UFD in delivering EBD to rodent brain. They reported that UFD was able to enhance drug volume distribution up to 2 times relative to CED^14^.

El Ghamrawy, A. et al. used a 5-MHz FUS transducer to study acoustic streaming in the tissue microenvironment. They injected a 0.1% bromophenol blue solution into three different soft tissue-mimicking gel and captured the movement of the solution with two cameras. They developed an experimental equation to estimate the average solution velocity. They reported the this average velocity in different ultrasound intensities^19^. In the later studies on the concept of drug penetration into the soft tissue (especially in the brain), Raghavan conducted a theoretical research to study the effect of acoustic streaming for drug delivery in soft tissue^20^. To the best of our knowledge, this was the first theoretical study that proposed an equation for drug penetration into the soft tissue porous microenvironment and claimed that the most important effect of acoustic waves on drug penetration is acoustic streaming. In order to derive the equations governing acoustic streaming in a porous medium, first, acoustic streaming of free fluid was evaluated, then acoustic streaming equations in the porous medium were derived. To investigate the ability of these equations in predicting experimental results, Raghavan compared exposure times of past studies with the results of analytical solutions of his equations. He reported that the results were promising, and the equation was able to predict the experimental results. However, acoustic sources were selected in a way that an analytical solution was easy to obtain.

Since the results of UCED were promising and most of the efforts conducted in UCED were preclinical studies, developing a model to simulate this process would be beneficial. This is particularly important when one notes that experimental efforts are high cost, and some crucial parameters may alter when an animal model is used instead of a human model. This point is further pronounced when considering that above 90% of the results obtained from animal experiments fail to predict the results in humans^21^, and figures are worse, up to 99.6% failure, in brain studies^22^. Moreover, because of the differences in inherent characteristics between human body and animal models, numerical models would be helpful since human-related parameters can be applied to them to eliminate the differences that exist between animal and human models. A numerical model that can also predict the effect of ultrasound waves on drug delivery will significantly decrease the cost of studies. Thus, it is necessary to investigate the ability of available equations in simulating the UCED process.

In this study, an experimental setup was developed to mimic the ultrasound-assisted drug delivery procedure. An array of ultrasound transducers with a resonance frequency of 1.1 MHz was used to focus the ultrasound waves. Agarose gels were synthesized to mimic the brain porous microenvironment, and drugs were injected into gels by a syringe pump. Then, the effect of FUS waves on pushing the drugs in the brain was studied in different exposure times and acoustic intensities. Along with the experiments, a finite element (FE) model was developed to simulate the only available equation in the literature and evaluate its ability to predict experimental results.

## Materials and methods

Since the aim of this study is to validate the results of the equation for acoustic streaming in the brain by the experimental data, we split this investigation into experimental and numerical parts. In the following, these two have been discussed.

### Experimental setup

#### Phased array transducers

Six disk transducers (Siansonic, Beijing, China) with a resonance frequency of 1 MHz were placed in a hemisphere shell annularly to produce FUS waves. The hemisphere shell radius was 10 cm, and the disk radius of each transducer was 10 mm (focal length = 10 cm, axial FWHM = 3 mm, lateral FWHM = 0.6 mm). Also, these transducers were 2 mm thick, with a maximum power 30 W per transducer. The physical properties of these transducers are listed in Supplementary Table 1.

#### Electrical Generator

An electrical generator (EMTco; Exon Electro-medical Technologies, Tehran, Iran) was utilized to produce 1-MHz sinusoidal ultrasound waves with up to 300 W electrical power. This generator allows the user to adjust the frequency and acoustic intensity while eliminating noise. Moreover, it uses a ramp function to ensure the transducers’ safety.

#### Brain-mimicking gel preparation

One of the most critical steps in UCED is the selection of background tissue for drug delivery. As mentioned, various tissues, including the equine brain, avian muscle, porcine brain tissue, rodent brain, and agarose gels have been used in the literature as the background tissue to mimic the properties of the human brain. Among these background tissues, 0.6-weight-percent agarose brain phantoms has an important advantage: it is easy to make, without costing a life. They are also transparent, which allows us to trace the drug distribution. Furthermore, it has been reported that they mimic well the neurological properties of human brain tissue, particularly in CED^15,18^.

The gels were prepared based on the recipe given in^15,18^. For preparation of 1X TBE buffer, first, 108 g of Tris was mixed with 55 g of Boric Acid. Next, 750 ml of dH2O was added and all three ingredients were mixed completely. Then, 40 ml of 0.5-M (7.5 g) EDTA Disodium was added. The flask containing the solution was filled to a final volume of 1 L by adding dH2O and it was stored at room temperature. Finally, a 1:10 dilution of the prepared TBE with dH2O created a 1X Working Solution. The buffer had a final pH of ˜8.3 and did not require adjustment.

Agarose gel was prepared using a weight/volume ratio of 0.6–0.7%. This optimal percentage of agarose would result in mimicking the brain microstructure efficiently^15^. Thus, 0.6 g of agarose powder was added to the flask containing 100 ml of TBE buffer. The agarose was allowed to sit in the solution for a few minutes before stirring. A stir bar and stirring plate were used to rapidly mix the solution. The flask was covered with a plastic wrap and a small hole was made in it to allow the solution to vent. The flask was heated in a household microwave for 30 s and then the solution was stirred again, gently. This process was repeated until a homogenous agarose solution was obtained. Then, the solution was cooled down to 55–60 °C and poured into a plastic container. The gel was then refrigerated for a few hours to solidify.

#### Drug injection system

The drug injection system included a syringe pump, a peripheral venous catheter, a connector tube, and a drug solution. Since the aim of this study is to investigate the effect of FUS waves on improving drug penetration in CED, the amount of drug solution and the volumetric flow injected into the background tissue must be determined so that the experiment can be reproducible in later studies. A syringe pump was utilized to inject 100 $\upmu$ L of drug solution at the volumetric flow of 60 $\upmu$ L/min via a peripheral venous catheter. Red food coloring was used to mimic the drug solution, allowing drug distribution tracking into the agarose phantom^18^. As the name of the connector tube suggests, it connects the syringe pump to the peripheral venous catheter.

#### Ultrasound-assisted convection-enhanced delivery

**Figure 2:**
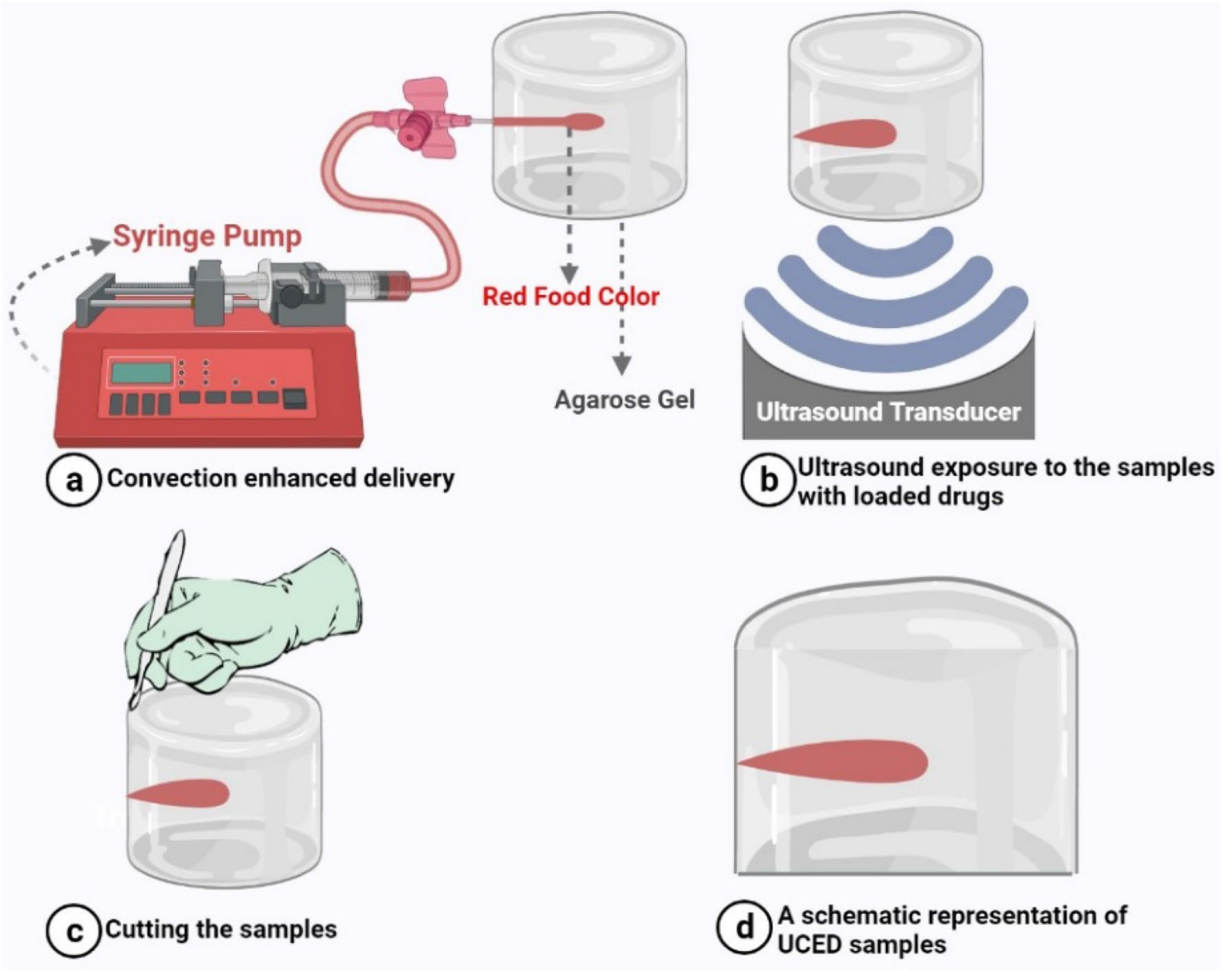
Schematic representation of UCED process. The drug solution is loaded in a syringe and is injected via a syringe pump to the brain mimicking gel (**a**). The peripheral venous catheter prevents potential damages caused by the movement of the tip of the catheter. After the drug solution is loaded, the focal zone of the phased array transducer is directed at the drug-injected area, and it is subjected to ultrasound waves (**b**). Right after the insonification by FUS waves, samples were cut to better determine the effect of these waves on drug delivery (**c**). (**d**) depicts a representation of samples exposed to UCED.

After preparing brain-mimicking gels and injecting drug solutions into the samples, they were exposed to FUS waves. Figure 2 shows a schematic of the UCED process. Four different experiments were conducted to better understand the effect of exposure time and intensity (spatial-peak temporal-peak intensity; ${I}_{SPTP}$) of FUS waves on drug penetration: One CED and three different UCEDs. Table 1 lists the acoustic intensities and exposure times used in these experiments.

**Table 1:** Details of the experiments.

Experiment	Acoustic intensity (W/cm^2^)	Exposure time (min)
I	6.72-9.67-13.20-17.20	30
II	13.20	20-30-40-50
III	17.20	20-30-40-50
CED	–	20-30-40-50

Experiment I was conducted at a constant exposure time with four different acoustic intensities. On the contrary, in Experiments II and III, the exposure time was changed from 20 to 50 min, and acoustic intensities were adjusted at 13.20 and 17.20, respectively.

### Acoustic streaming and drug penetration depth estimation

In this section, first, ultrasound waves generated by an FUS transducer were obtained. Then, by assuming the brain tissue as a porous medium (with low permeability), acoustic streaming equations were solved. An FE model was developed to evaluate the ability of the acoustic-streaming equation in porous media in predicting experimental results. To develop this FE model, we used COMSOL Multiphysics which is proven to combine and solve problems with different physical equations and is widely used in biomedical applications of ultrasonic waves^23–26^.

#### Ultrasound waves propagation calculation

In this study, a spherical 2D-axisymmetric transducer with a focal length of 50 mm and a resonance frequency of 1.1 MHz was used as the FUS source. The frequency was set at 1.1 MHz to be validated with the data in the literature and at the same time be close to the experimental value (1 MHz) with adequate precision. In order to obtain ultrasound field variables such as pressure and velocity, the Helmholtz equation must be solved. This equation describes the propagation of sound waves with attenuation in a given medium in the frequency domain^27^:

1 $$\nabla .\left(-\frac{1}{{\rho }_{c}}\nabla p\right)-\frac{{k}_{eq}^{2}p}{{\rho }_{c}}=0.$$

In the above equation, $p$ is the ultrasound pressure. Furthermore, equivalent wave number (${k}_{eq}$) and complex density (${\rho }_{c}$) are defined as^27^:

2 $${k}_{eq}^{2}={\left(\frac{\omega }{c}-i\alpha \right)}^{2},$$

3 $${\rho }_{c}=\frac{\rho {c}^{2}}{{\left(\frac{\omega }{\frac{\omega }{c}-i\alpha }\right)}^{2}},$$

in which $\alpha$ is the attenuation coefficient and $\rho$, $\omega$ and $c$ denote the density, angular frequency, and sound speed, respectively.

The acoustic properties of brain-mimicking gels are presented in Supplementary Table 2. Given the fact that brain-mimicking gels are symmetrical about the propagation line of the ultrasound waves, a 2D-axisymmetric FE model was developed to simulate UCED.

**Figure 3:**
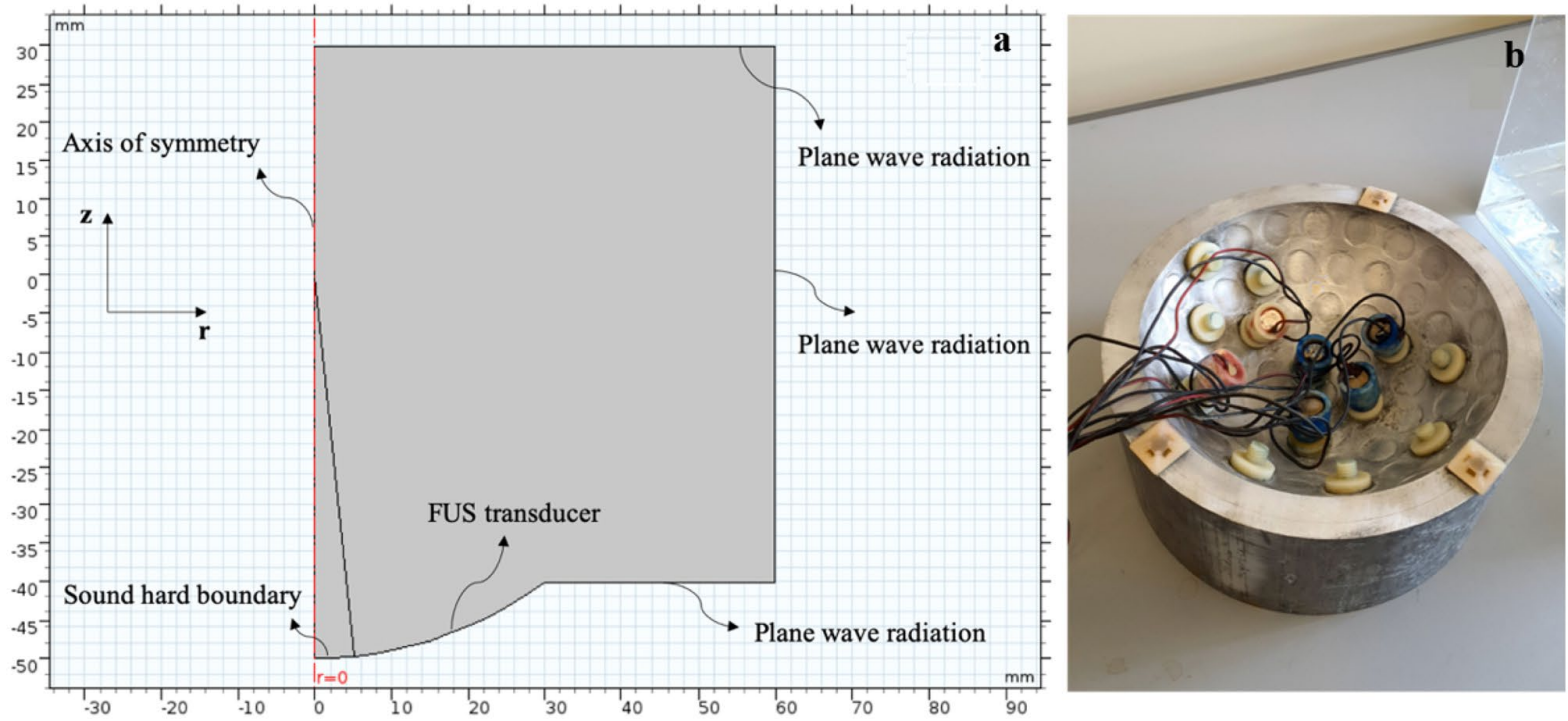
**(a)** The cylindrical domain of the numerical model and the boundary conditions. The red dashed line denotes the axis of symmetry. Upon the revolution of the geometry around the axis of symmetry, the model becomes three-dimensional. **(b)** The FUS transducer used to generate acoustic streaming for convection enhanced delivery. Six disk transducers with a resonance frequency of 1 MHz were placed in a hemisphere shell annularly to produce FUS waves. The hemisphere shell radius was 10 cm, and the disk radius of each of the transducers was 10 mm with a 2-mm thickness and 30 W power per transducer.

In the model, in order to generate ultrasound waves, a normal displacement was applied to the transducer boundary. Other boundaries were assumed to be plane wave radiation boundaries which allow sound beams to pass through without any reflection. Figure 3a shows the geometry and boundary conditions of the FUS transducer, which can be compared with the transducers used in the experiments, as shown in Fig. 3b. It is worth mentioning that the ultrasound domain after revolution around the axis of symmetry would be a cylinder which can be a good representation of the experimental environment.

#### Acoustic streaming in porous media

While, various studies have been conducted to investigate the acoustic streaming effect, only one study has proposed acoustic streaming equations in a porous medium. This equation, proposed by Raghavan for describing streaming velocity and pressure, is as follows^20^:

4 $$\gamma u=-\nabla p-\rho \nabla .\left(v.v\right),$$

5 $${\nabla }^{2}p=\nabla .\left(-\rho \nabla .\left(v.v\right)+\frac{\varepsilon \gamma }{\rho }\left({\rho }_{1}v\right)\right),$$

Here, $p$,$v$, and ${\rho }_{1}$ are respectively the pressure, velocity, and density of sound. The porosity of tissue is defined by the unitless parameter $\upvarepsilon$, and $\upgamma$ can be expressed as:

6 $$\gamma =\frac{1}{K},$$

where $K$ is the hydraulic conductivity.

### Safety considerations

While ultrasound waves are a promising tool for improving drug delivery efficiency and can significantly reduce drug side effects, safety issues in ultrasound as an imaging or therapeutic tool must be considered. One of the simplest ways to define limitation guidelines for the safety of a ultrasound system is the use of mechanical index (MI). This unitless number creates a dividing line between circumstances where cavitation initiates and the situation where no or little steady vibrations of bubbles are present. These bubbles either exist in the medium or they can be generated when ultrasound waves are applied onto the human body. MI can be determined with the formulation below^17,28^:

7 $$MI=\frac{{P}_{n}}{\sqrt{f}},$$

where ${P}_{n}$ denotes peak negative pressure in MPa and $f$ is the frequency of the waves in MHz. In this study, the maximum pressure corresponds to Experiment III. From the simulations and under the conditions of Experiment III, the peak negative pressure is 0.8 MPa, and the resonance frequency of the source is 1.1 MHz. Therefore, MI = 0.76 is the maximum value that occurs in our experiments. The safety threshold is usually considered MI = 1.9^28^. Therefore, in the view of MI, the parameters of our setup are safe even for use in the human brain and have pre-clinical value.

## Validation

Since Eq. (4) and (5) give acoustic streaming velocity in a porous medium and the experimental studies reported the drug penetration depths before these equations were reported, Raghavan validated his equation using exposure times. He extracted the penetration depths from figures or tables in the past studies and calculated the exposure time by the following integration^20^:

8 $$T\left(R\right)=\phi {\int }_{{\varepsilon }_{0}}^{R}\frac{dr}{v(r)},$$

where $\phi$ is the porosity, $v(r)$ is the velocity of the liquid, $T$ is the exposure time, and $r$ is the radial distance (in the spherical coordinate). Finally, he compared the calculated exposure times with prior experiments. Using this method of validation, however, diverse results were obtained. In other words, some exposure times were close to those in the experiments, and some did not match experiment data.

**Figure 4:**
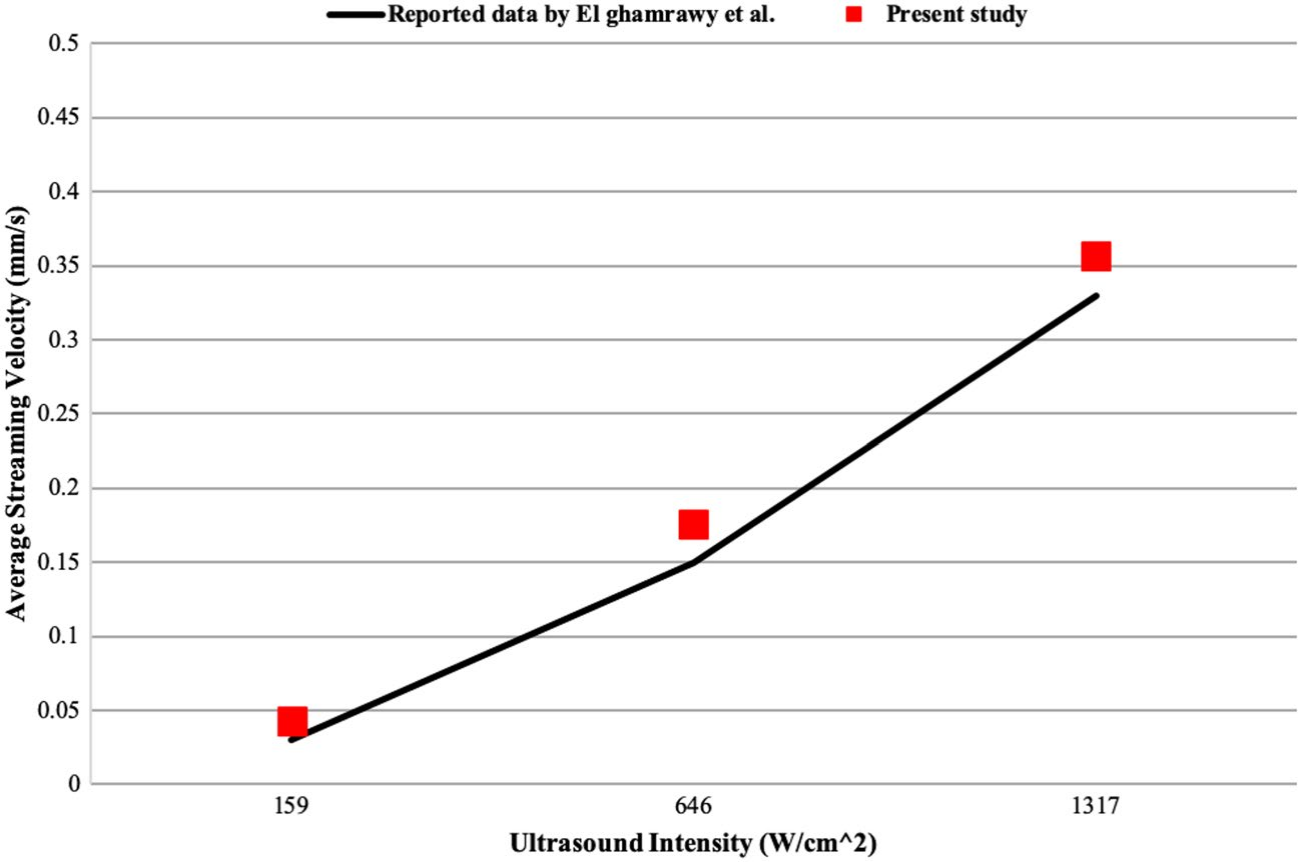
Validation of the numerical method by calculating average streaming velocities. The continuous line represents the results of El-ghamrawy et al.^29^, and the red squares denote the results of the present study.

Given the fact that El-ghamrawy et al.^29^ reported average streaming velocities at different acoustic intensities, a different validation approach was considered in the study at hand. In this approach, first, acoustic pressure and velocity were extracted by solving Eq. (1) and setting maximum acoustic intensities and acoustic parameters on what El-ghamrawy et al. reported (Supplementary Fig. 1)^29^. Second, streaming velocity profiles were calculated from Eq. (5) and (6). Eventually, average streaming velocities were obtained. As Fig. 4 illustrates, a good agreement exists between findings of this study and the data reported in^19^ as the difference between the two methods in average streaming velocities are 44.33%, 17.33% and 8.18% for 159 W/cm^2^, 646 W/cm^2^ and 1317 W/cm^2^ ultrasound intensities, respectively, relative to the data from literature.

## Results and discussion

In this section, the results of the experiment and numerical parts are reported. First, the effect of ultrasound waves on enhancing the drug solution penetration through the gels is reported, then these results are quantified, and at last the drug volume distribution of each sample is presented. Second, the ultrasound intensities are adjusted to the values of the experiments, and the streaming velocities are calculated. Finally, the drug penetration depths from the experiments and the simulations are compared, and the ability of the FE model in predicting the empirical results is discussed.

### UCED in brain-mimicking gels

**Figure 5:**
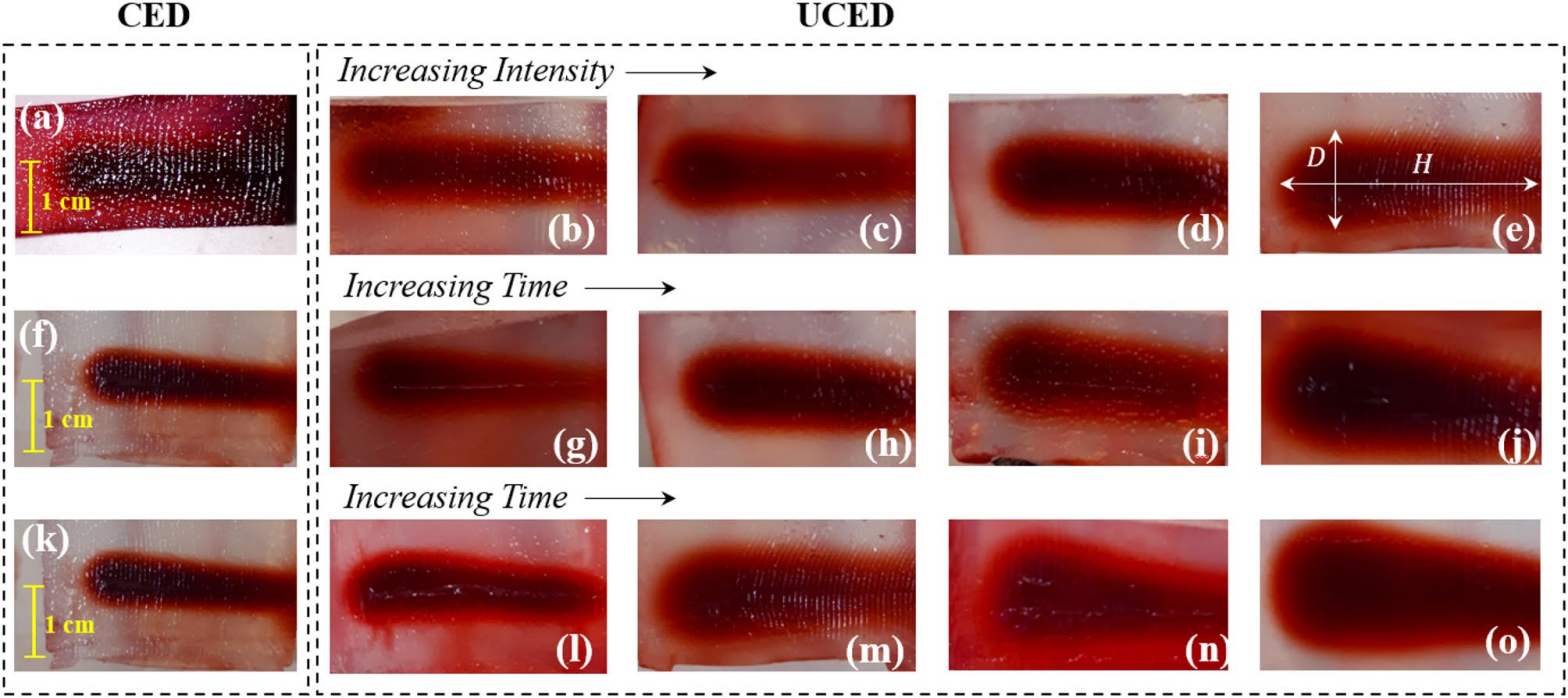
Drug distribution over the mid-surface of the brain-mimicking gels: The first row (**a-e**) depicts the variations of drug distribution over different ultrasound intensities (Experiment I). In this row, the left image depicts the CED sample after 30 min exposure time and the remaining four figures show the outcome of Experiment I, where ultrasound intensity changes according to the values in Table 1, from left to right In the second (**f-j**) and third row (**k–o**), the left image shows the CED sample’s result after 50 min exposure time and other four figures in each row are the results of Experiments II and III, showing variations of drug distribution with the increase of time, according to the values in Table 1. Subfigure (**e**) shows how the volume of the penetrated drug was calculated by a cylindrical calculation from parameters *D* and *H*.

**Figure 6:**
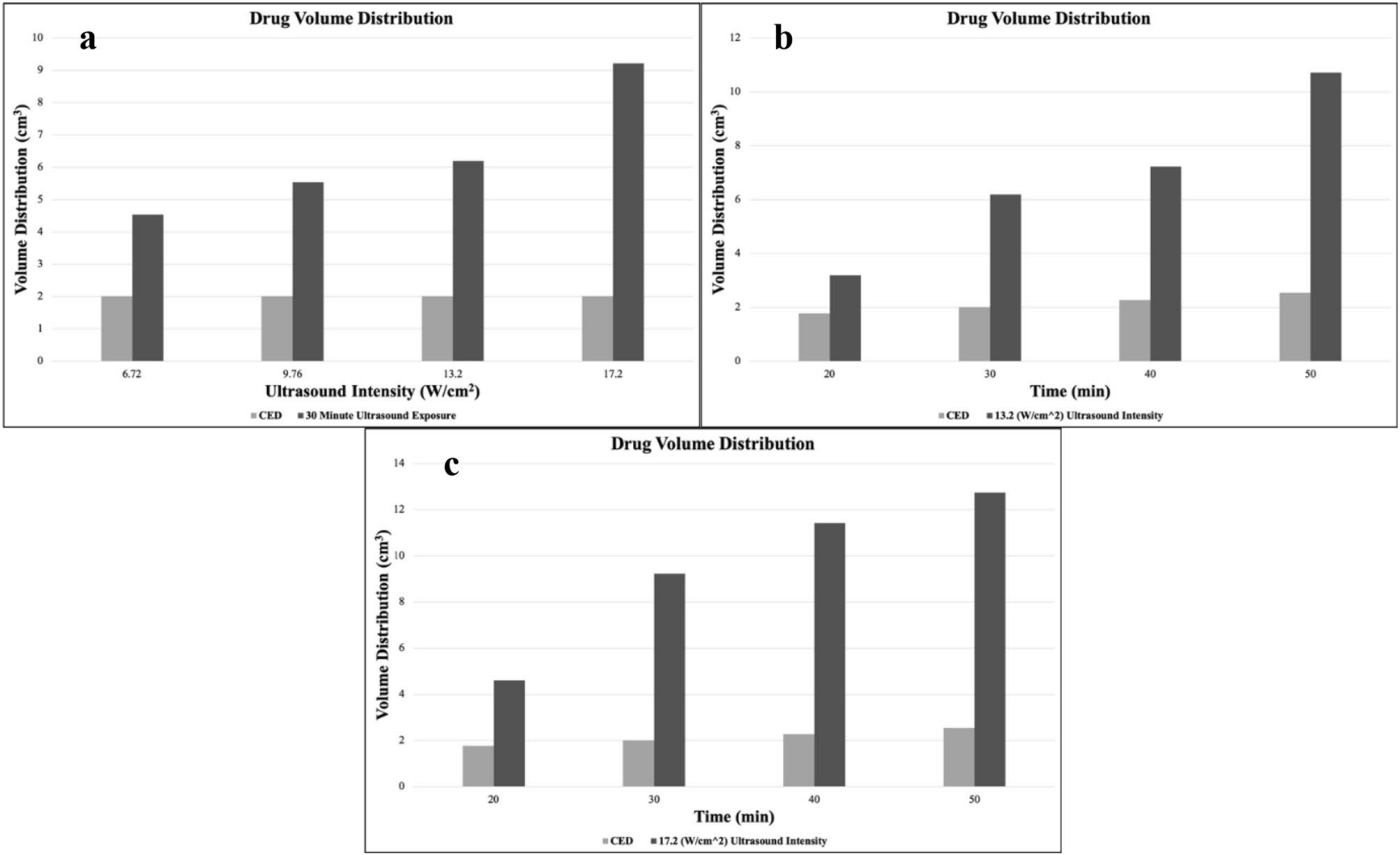
Variations of drug volume distributions for the three experiments: (**a**) shows the values for Experiment I (dark gray data) and CED samples (light gray data) for 30 min of ultrasound exposure at different ultrasound intensities. (**b**) shows the increase in drug volume distribution over time for Experiment II (dark gray data) at an intensity value of 13.2 W/cm^2^, compared with CED results (light gray data) (**c**) indicates the comparison between the drug volume distributions calculated from the data of Experiment III (dark gray data) and from CED samples (light gray data) with different exposure times at an intensity value of 17.2 W/cm^2^.

Right after the experiments (Table 1), each sample was cut at the middle surface, as shown in Fig. 5a–o. As it is evident in this figure, FUS waves improve the drug distribution through the gels, and as the exposure time and the ultrasound intensity increase, the drug penetration depth (*D*) improves relative to CED. To better show the impact of ultrasound waves on drug distribution, CED samples are reported in the left figures of each row in Fig. 5. In order to better analyze the results of UCED in enhancing the drug delivery, a cylindrical distribution pattern was assumed, and the volume of each sample was approximated by $$\,\overset{\lower0.5em\hbox{$\smash{\scriptscriptstyle\smile}$}}{V} = \pi D^{2} H/4$$, where $$D$$ and $$H$$ are respectively the diameter and height of the penetrated solution, as shown in Fig. 5e. After quantifying the results of experiments, a better judgment can be made. From Fig. 6a, it can be concluded that the ultrasound waves have enhanced the drug volume distribution by about 226%, 275.7%, 308%, and 459% relative to CED, with the increase of ultrasound intensity from 6.72 to 9.67, 13.2. and then 17.20 W/cm^2^ (Experiment I), respectively, within the exposure time of 30 min.

In order to assess the impact of exposure time of the ultrasound waves in UCED, Experiments II and III were designed, where ultrasound intensity is fixed (at two values of 13.20 W/cm^2^ and 17.20 W/cm^2^), while exposure time is varied. In contrast, in Experiment I, the exposure time was constant, and consequently the CED data remained almost constant. As shown in Fig. 6b,c, the change in the CED results (gray) with time is very slow. In other words, time had a relatively small effect on improving the outcomes of CED. However, time had a significant effect on the UCED drug volume distributions. That is, in Experiment II, by increasing the time from 40 to 50 min, drug volume distribution grew 48%, but this number was about 12% for CED, which shows the effectiveness of the UCED method.

Since the only force for propagating drug solution into brain tumor in CED after cutting the syringe pump (or pressure) is diffusion, it is no surprise that the drug volume distribution of the CED samples grew slowly during the specified time. Moreover, it is worth mentioning that in Experiment II, 180%, 308%, 318% and 421% enhancement was made by using UCED, when compared to CED, for exposure times of 20, 30, 40 and 50 min, respectively. These values were 260%, 459%, 503% and 500% for Experiment III. In clinical practice, this improvement in drug volume distribution could translate to better drug delivery to the brain tumors and, hopefully, effective treatment of these tumors.

While it has been reported that the increase in time and ultrasound intensity lead to better drug delivery and more drug volume distribution through brain-mimicking gels, it must be noted that there is always an upper limit for exposure time and ultrasound intensity since as time and intensity grow the thermal effects of FUS waves become stronger and may lead to necrosis of the healthy brain tissue. Furthermore, one can claim that it is true that it takes time for CED results to reach the outcomes of the UCED and if we give CED enough time in the scale of days to CED, it can replicate the results of UCED. This claim is valid for a passive background of drug delivery, but, in a real brain with the existence of circulatory system and other complex organs, the major amount of drug solution dissipates through the clearance into capillaries and brain tissue, and thus, the CED cannot replicate the efficiency of UCED^16,30^.

Another possible scenario can be extending the time of applying pressure and continuing the infusion, until the distribution of drug reaches a desired value. While prolonging the infusion can greatly impact the efficiency of drug delivery, sometimes this process can take hours and this duration is infeasible^17^. In any case, the main focus of this paper is not to contradict CED but to make it more feasible and quicker.

### Comparison between the numerical model and the experiments

As discussed in the earlier sections, there is only one equation describing the acoustic streaming in a porous medium proposed by Raghavan^20^. This equation describes the multi-dimensional acoustic streaming for soft, porous materials and does not contain any terms for the circulatory system or other scenarios in a real brain. However, since we used brain-mimicking gels that are inherently passive in this study, this equation is appropriate for developing a numerical model of this drug delivery method. To achieve this goal, first, the ultrasound intensities were set at the numbers used in the experiments (Supplementary Fig. 2), and ultrasound pressures were calculated (Eq. (1)). Then, these pressures were assumed as the inputs of Eq. (4) and (5). As there is no pre-defined physics for Eq. (4) and (5), two Coefficient-Form PDEs were developed in COMSOL to obtain streaming velocity and pressure. Since the effect of waves on producing streaming velocities is much more significant in the axis of symmetry of the FUS transducer, other directions are assumed to have zero contribution in streaming^31^.

**Figure 7:**
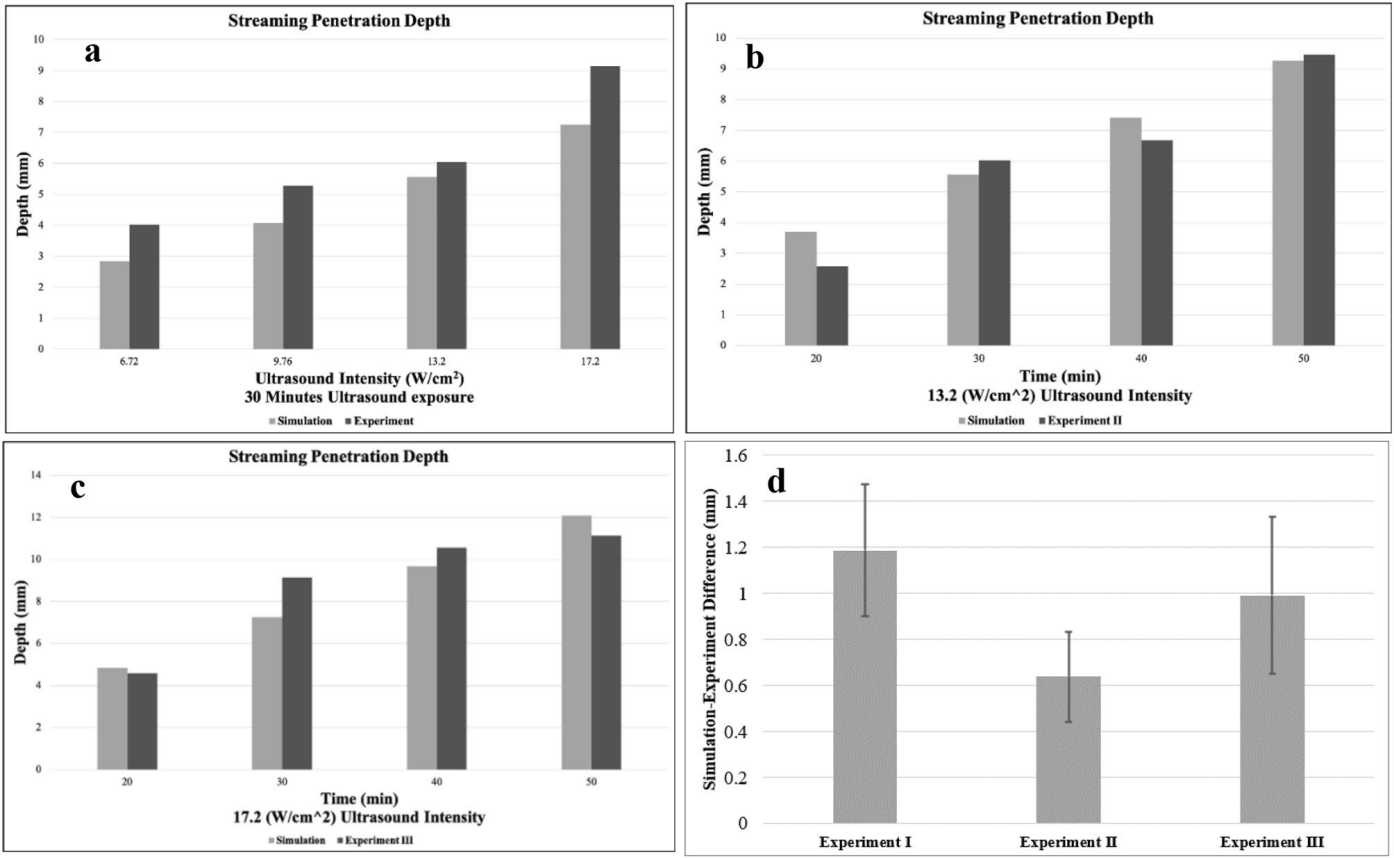
A comparison between drug penetration depths obtained from experiments and FE model for Experiment I, II and III is shown in (**a**), (**b**) and (**c**), respectively. The drug penetration depths in the numerical models were calculated by multiplying average velocities by exposure times. The mean difference between the numerical and experimental results, alongside with standard errors are shown in (**d**).

By solving Eq. (4) and (5), streaming velocity profiles were obtained, as displayed in Supplementary Fig. 3. Next, the average streaming velocities were calculated. These average velocities were multiplied by the exposure time of each experiment to obtain the drug penetration depths. To better compare the drug penetration depths of the numerical model and experimental setup, these values are shown in Fig. 7a,b,c for Experiments I, II and III, respectively. These data show that Eq. (4) and (5) produce results that match the experimental results in all three experiments with a slight difference. While the difference between numerical and experimental results may vary for each experiment, for most of them, the mean error remained under 23% (Table 2), which shows the ability of this numerical model to replicate the experimental data. When outlier data (> 30%) is removed, the mean value of simulation-experiment difference is 20.11 ± 8.92%, 7.01 ± 4.52%, 10.69 ± 6.84% for Experiments I to III, respectively, when the difference percentage is calculated relative to the experimental data as reference. Figure 7d reports the mean value of the simulation-experiment difference, alongside the standard error bar, showing a good agreement between numerical and experimental results in a collective sense. This validation method fills the gap between experimental efforts and results from solving the equation proposed by Raghavan^20^. Further, the results are consistent for all the cases, in contrast to the diversity reported in^20^ for validating the results of UCED using Eq. (7). It is worth mentioning that most of the simulation penetration depths are smaller than the results of experiments. This difference may be interpreted as the thermal effects of ultrasound waves that can make little change in the microstructure of the brain-mimicking gels. Evidence for this is the increase in the simulation-experiment difference at the highest intensity, i.e. 17.2 W/cm^2^ in Experiment I (Fig. 7a), where heating is expected to be the most.

**Table 2:** This table shows the errors of numerical penetration depths compared to each experiment.

Error	**Experiment I**		6.72 W/cm^2^	9.76 W/cm^2^	13.2 W/cm^2^	17.2 W/cm^2^	Mean difference $\pm$ SD	
	Intensity							
Absolute difference (mm)			1.176	1.194	0.478	1.89	1.187 $\pm$ 0.572 (mm)	
Difference (%)			29.25	22.61	7.91	20.68	20.11 $\pm$ 8.92 (%)	
Error		Time	20 min	30 min	40 min	50 min	Mean difference $\pm$ SD	
**Experiment II**								
Absolute difference (mm)			1.128	0.478	0.736	0.2	0.638 $\pm$ 0.393 (mm)	
Difference (%)			43.72	7.91	11.02	2.11	16.91 $\pm$ 18.72 (%) [7.01 $\pm$ 4.52 (%) excluding the outlier]	
**Experiment III**								
Absolute difference (mm)			0.236	1.89	0.888	0.95	0.991 $\pm$ 0.681 (mm)	
Difference (%)			5.13	20.68	8.41	8.53	10.69 $\pm$ 6.84 (%)	

The first three rows are data related to Experiment I, and others are error data for Experiment II and Experiment III. The relative differences, with experimental data as the reference, are listed as well.

## Conclusion

This paper is written in two parts. First, the phased-array transducer of one of our previous studies was modified and redesigned to produce ultrasound waves for UCED. Then, the CED process was conducted, and after loading a drug solution into several brain mimicking gels, the samples were exposed to ultrasound waves with discrete increases in intensity and exposure time. The outcomes of the experimental part showed that ultrasound waves could enhance the drug volume distribution by up to 500% relative to the CED results. Second, a numerical FE model of the UCED process was developed to assess the performance of the only existing equation for describing acoustic streaming—the primary effect of ultrasound waves in UCED—in a porous medium. For this purpose, we validated our method with two different approaches: first, we validated the average streaming velocities with the data of El-ghamrawy et al. and then we validated our obtained penetration depths to those obtained from the experiments. Our results indicated that the results from Raghavan’s equation are in good agreement with experimental outcomes. Moreover, there was a good agreement between simulation and experimental results, with less than 21% mean difference, when the outlier data was excluded. 

In recent years, targeted drug delivery methods have significantly improved drug delivery efficiency with various disciplines, including chemical, electrical, and mechanical tools. Among these tools, ultrasound waves have shown promising outcomes in enhancing the treatment efficiency. However, most of these efforts are at their primary steps and require many investigations to disclose and measure every parameter that affects such methods. While clinical studies can be a significant step toward understanding these methods, they are high-cost, cannot control some parameters, and have ethical issues. Regarding these disadvantages, developing finite element models are valuable because they are low-cost, can be patient-specific, and can easily control the geometry and physical parameters. Also, the mechanical properties of human tissue can be used in them for more realistic models. The aim of this study was to validate the experimental outcomes with a FE model directly. This approach can be used to build more complex models of the human brain with a tumor and other live organs to better understand the effect of ultrasound waves in drug delivery to tumors.

## Supplementary Information

Supplementary Information.

## Data Availability

All data generated or analyzed during this study are included in this published article (and its Supplementary Information files).
