# Supplementary material for: Numerical and experimental evaluation of ultrasound-assisted convection enhanced delivery to transfer drugs into brain tumors
Source: Sci Rep. 2022 Nov 11;12:19299. doi: 10.1038/s41598-022-23429-w (PMC9652304; doi:10.1038/s41598-022-23429-w)
Supplement: Supplementary file 1 — Supplementary Information. [file 41598_2022_23429_MOESM1_ESM.docx]

**Supplementary Materials**

**Figure S1:**

**
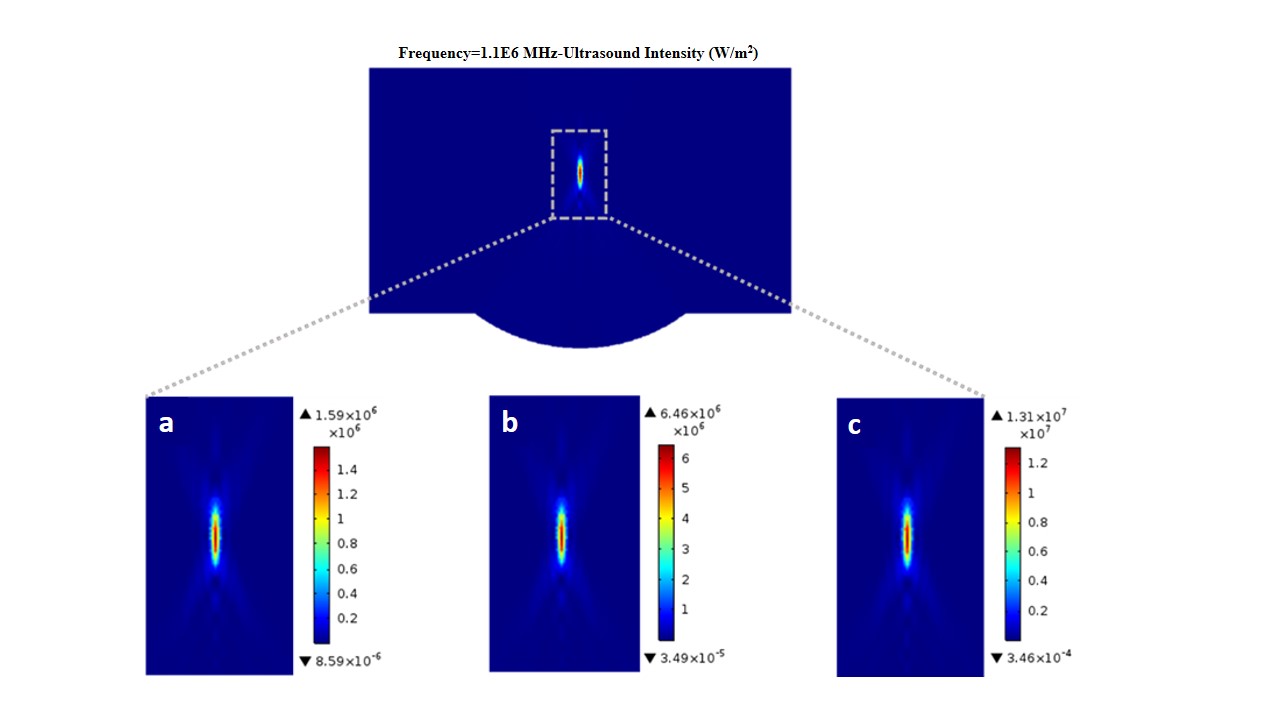
**

**Figure S2:**

**
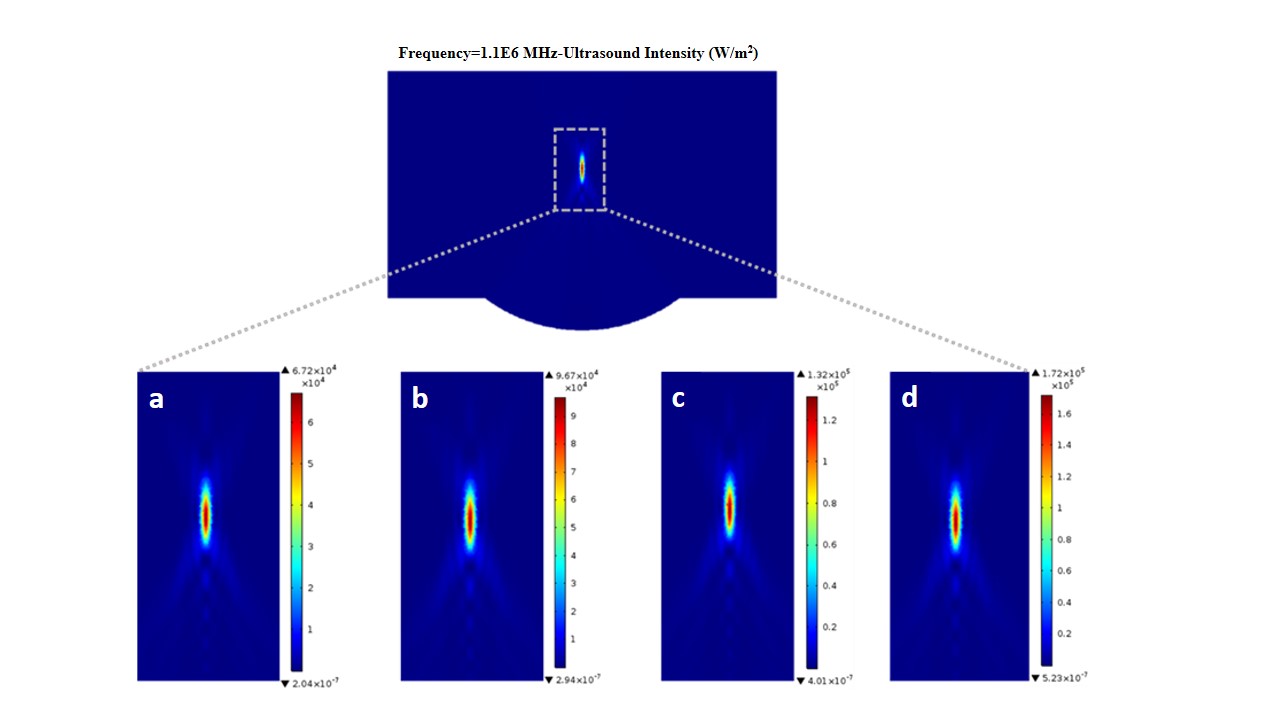
**

**Figure S3:**

**
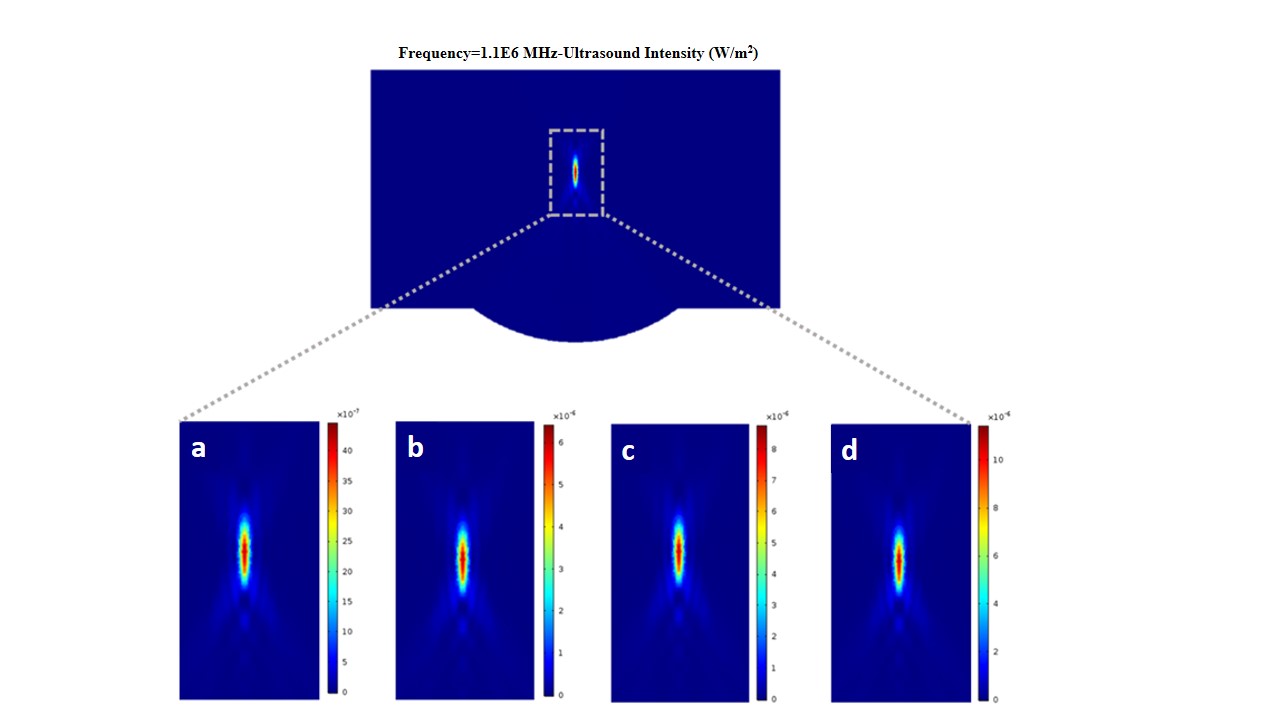
**

| Table S1   \| Frequency \| $1 MHz$ \| \| --- \| --- \| \| Internal Resistance ($R_{0}$) \| $\leq2 \Omega$ \| \| Internal capacitance ($C_{p})$ \| $1nf\pm30\%$ \| \| Thickness piezoelectric coefficient ($d33$) \| $\geq300 Pc/N$ \| \| Weight \| $1.443 g$ \| |
| --- | --- | --- | --- | --- | --- | --- | --- | --- | --- | --- |
| Table S2 |

| **Parameter** | **Value** |
| --- | --- |
| Attenuation coefficient ($\alpha$) | 0.16 $1/cm$ |
| Density ($\rho$) | 1000 $kg/m^{3}$ |
| Speed of sound ($c$) | 1600 $m/s$ |
| Hydraulic conductivity ($K$) | 2×10^-10^ $m^{4}/N$ |
| Porosity ($\varepsilon$) | 0.2 |

**Supplementary Figure legends:**

**Supplementary Figure 1.** Ultrasound intensity profiles. The maximum values were adjusted to match the values reported by El ghamrawy et al ^25^, i.e., 159, 646, 1317 W/cm^2^

**Supplementary Figure 2.** Ultrasound intensity profiles. The maximum values were adjusted to match the values of the experiments.

**Supplementary Figure 3.** Acoustic streaming profiles for each FUS strength applied in this study. (**a**), (**b**), (**c**), and (**d**) are the results of acoustic streaming for ultrasound intensities of 6.72, 9.76, 13.20, and 17.20 W/cm^2^, respectively.

**Supplementary Table legends:**

**Supplementary Table 1.** Detailed characteristics of each of the disk transducers

**Supplementary Table 2.** Acoustic properties of tissue and drug. The speed of sound, density, and attenuation coefficient of the drug solution and the agarose gel are assumed to be identical.
